# A Rare Case of Radial Arteriovenous Fistula (AVF) Post-Transradial Percutaneous Coronary Intervention

**DOI:** 10.7759/cureus.54943

**Published:** 2024-02-26

**Authors:** Dibyasundar Mahanta, Sindhu Rao Malla, Saran P Mohanan, Pranjit Deb, Debasish Das

**Affiliations:** 1 Cardiology, SUM Hospital, Bhubaneswar, IND; 2 Cardiology, All India Institute of Medical Sciences, Bhubaneswar, Bhubaneswar, IND

**Keywords:** compression, inadequate, paying, arterio-venous fistula, radial

## Abstract

We report a rare case of radial arteriovenous fistula in a middle-aged person after a successful transradial percutaneous coronary intervention. Early release of manual radial compression was the culprit behind the development of radial arteriovenous fistula. Early surgical repair of the radial arteriovenous fistula resulted in the complete resolution of distal forearm symptoms in the abovementioned patient. It is advised for young interventional cardiologists to apply a compressive bandage over the radial artery for a minimum period of one hour to get rid of this extremely rare complication.

## Introduction

Transradial coronary access has limited complications for which it is favored worldwide as the preferred site access for coronary intervention [1]. Transradial coronary access is relatively safe as compared to transfemoral access as transfemoral access sometimes gets complicated by the development of local hematoma, femoral arteriovenous fistula (AVF), and life-threatening retroperitoneal bleed. Lower femoral puncture carries a risk of formation of femoral pseudoaneurysm while higher transfemoral access above the inguinal crease carries the risk of retroperitoneal hematoma. The beauty of transradial access lies in achieving rapid hemostasis with manual compression. The most common complication of transradial access is radial artery spasm followed by radial artery dissection and thrombosis of the radial artery resulting in loss of radial pulse post-coronary intervention. A radial AVF is an extremely rare complication of transradial access as if at all the puncturing needle crosses through the lumen of the radial artery and vein, it gets sealed off with subsequent compression either with a bandage or with a radial band. We describe an extremely rare case of radial AVF post-coronary intervention due to inadequate compression which was surgically ligated long after coronary intervention. Application of compressive bandage over the radial puncture site for a minimum period of one-hour post-procedure with compressive bandage in place for a minimum period of 24 hours post-procedure by young interventional cardiologists can prevent such rare complications.

## Case presentation

**Figure 1:**
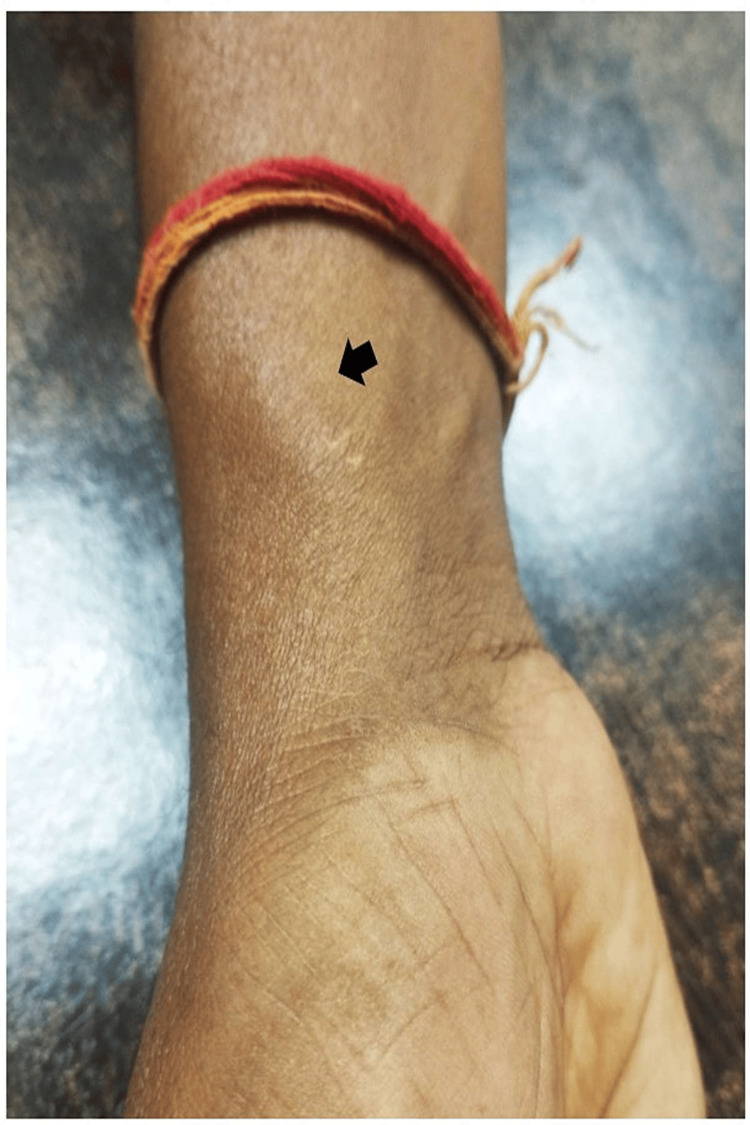
Pulsatile swelling near the radial access site (radial arteriovenous fistula)

**Figure 2:**
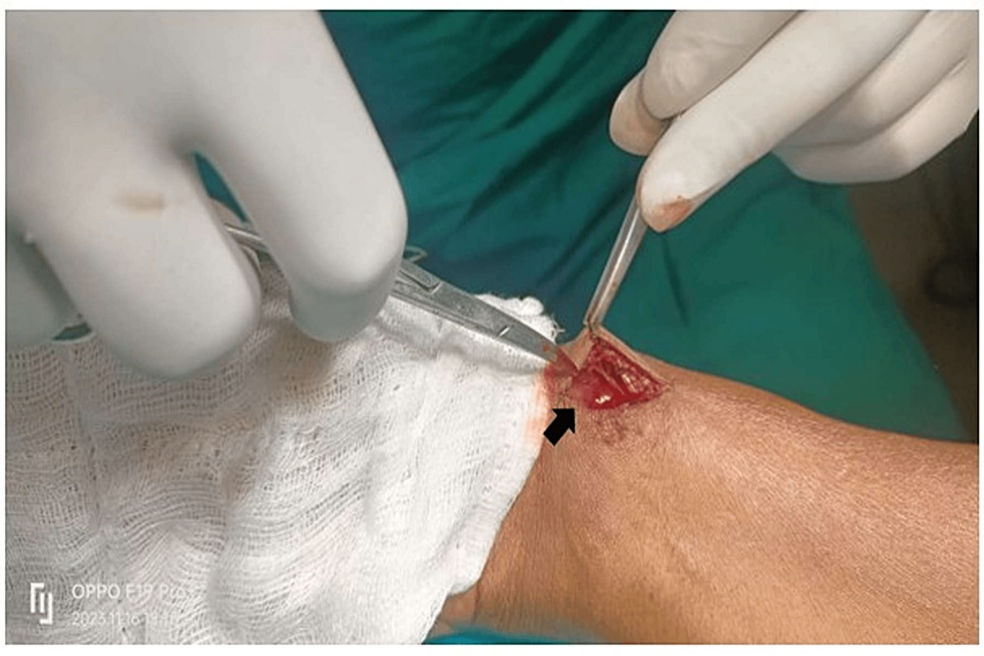
Arterialized vein (which has grown in size) in radial arteriovenous fistula

A 45-year-old male nondiabetic, nonhypertensive, smoker, and alcoholic presented to the cardiology outpatient department 6 months after his transradial right coronary angioplasty. He did not have any angina or shortness of breath post-coronary intervention. He was normotensive and his ECG did not reveal any fresh ischemic changes. He complained of resting palpitation most of the time and had an unusually warm sensation with pain in the distal forearm. He complained of a pulsatile swelling at the radial access site (Figure 1). Local site examination revealed a to-and-fro murmur of radial AVF. Due to arterial runoff, he had a pounding sensation in the heart due to a hypervolemic hyperdynamic status in the radial AVF. Doppler interrogation revealed a large radial AVF. As the radial AVF presented after 6 months post-coronary intervention, we did not opt for ultrasound-guided compression of the radial AVF. He was sent for surgical repair of radial AVF. Under local anesthesia, a one-inch incision was given over the radial styloid process and the subcutaneous fascia was dissected. The arterialized radial vein was visible which was highly pulsatile secondary to radial AVF and the fistulous connection was ligated (Figure 2). The skin was sutured and the patient was discharged the same day after ligation of the radial AVF. He was doing well in follow-up, there was no murmur at the radial access site, and Doppler flow was laminar. The patient remembered the incident as the duty resident had loosened the compression bandage over the radial puncture site 15 minutes after the procedure as he had severe ischemic pain and swelling of fingers due to tight radial artery compression with the bandage. Early release of the compressive bandage over the radial puncture site resulted in persistent radial AVF with hypervolemic and hyperdynamic circulation. Adequate and prolonged radial artery compression with a compressive bandage over the radial puncture site for a minimum period of 24 hours can prevent such extremely rare radial AVF in post-coronary intervention patients. As small mistakes in the coronary intervention are usually paid off, we paid for an inadequate compression of the radial artery with the development of delayed radial AVF.

## Discussion

Common complications of transradial coronary intervention include radial artery spasm, radial artery hematoma, and diminished or absent radial pulse. AVF occurs commonly secondary to the femoral or brachial access, but it is an extremely rare complication after transradial intervention [1]. This is because small veins are present around the radial artery. There are known risk factors for radial AVF, but ultrasound-guided radial artery cannulation may minimize the risk of radial AVF. In hemodynamically nonsignificant shunt and asymptomatic persons, radial AVF is managed conservatively. For symptomatic radial AVF, percutaneous closure of the fistula and surgical correction are the treatment modalities [2]. Early and adequate compression of the radial artery seals and prevents the development of radial AVF. The compressive bandage in the index patient was removed after 15 minutes as the patient was having excruciating pain in the palm and fingers post-transradial coronary intervention. Patients with radial AVF present with palpable thrill, bruit, and pulsatile mass. The index patient had warmth and pain in the distal forearm secondary to a radial AVF. Doppler ultrasound distinguishes radial AVF from hemangioma, pseudoaneurysm, and malignant tumors [3]. CT angiography delineates the relation between the radial artery, vein, and adjacent structures around the AVF. Due to the small size of the radial artery, percutaneous intervention has a limited role in the management of radial AVF. Summaria et al. did the first percutaneous closure of radial AVF after failed ultrasound-guided compression [4]. Sugahara et al. reported a method of balloon-assisted percutaneous embolization in AVF who refused surgical ligation [5]. The index patient was not subjected to percutaneous intervention as the patient denied the same and the radial AVF was surgically ligated which was a minor surgical procedure and the patient was discharged home on the same day. During follow-up after 7 days, he had no palpable thrill or audible bruit, he had no warmth sensation or pain in the distal forearm and the vascular Doppler ultrasound was within normal limit.

## Conclusions

We report a rare case of radial AVF in a middle-aged person secondary to inadequate compression of the radial artery. Although femoral AVF in low femoral arterial puncture is a quite common complication, radial AVF is an extremely rare complication encountered in routine clinical practice. Early adequate compression with compressive bandage, ultrasound-guided compression, and surgical correction are the modalities to treat radial AVF. Young interventional cardiologists should give attention to adequate compression of the radial artery with the application of a compressive bandage post-intervention for a minimum period of one hour to get rid of such unusual complications.
